# Label‐free quantitative analysis of the casein kinase 2‐responsive phosphoproteome of the marine minimal model species *Ostreococcus tauri*

**DOI:** 10.1002/pmic.201500086

**Published:** 2015-06-09

**Authors:** Thierry Le Bihan, Matthew Hindle, Sarah F. Martin, Martin E. Barrios‐Llerena, Johanna Krahmer, Katalin Kis, Andrew J. Millar, Gerben van Ooijen

**Affiliations:** ^1^ School of Biological SciencesUniversity of EdinburghEdinburghUK

**Keywords:** Casein kinase 2, Cell biology, Circadian clock, Marine algae, *Ostreococcus tauri*, Phosphoproteomics

## Abstract

Casein kinase 2 (CK2) is a protein kinase that phosphorylates a plethora of cellular target proteins involved in processes including DNA repair, cell cycle control, and circadian timekeeping. CK2 is functionally conserved across eukaryotes, although the substrate proteins identified in a range of complex tissues are often different. The marine alga *Ostreococcus tauri* is a unicellular eukaryotic model organism ideally suited to efficiently study generic roles of CK2 in the cellular circadian clock. Overexpression of CK2 leads to a slow circadian rhythm, verifying functional conservation of CK2 in timekeeping. The proteome was analysed in wild‐type and CK2‐overexpressing algae at dawn and dusk, revealing that differential abundance of the global proteome across the day is largely unaffected by overexpression. However, CK2 activity contributed more strongly to timekeeping at dusk than at dawn. The phosphoproteome of a CK2 overexpression line and cells treated with CK2 inhibitor was therefore analysed and compared to control cells at dusk. We report an extensive catalogue of 447 unique CK2‐responsive differential phosphopeptide motifs to inform future studies into CK2 activity in the circadian clock of more complex tissues. All MS data have been deposited in the ProteomeXchange with identifier PXD000975 (http://proteomecentral.proteomexchange.org/dataset/PXD000975).

**Abbreviations**
ACN: acetonitrile
CCA1: circadian clock‐associated 1
CK1: casein kinase 1
CK2: casein kinase 2
DHB: 2,5‐dihydroxybenzoic acid
FDR: false discovery rate
FRQ: frequency
LD: light/dark
LL: constant light
LUC: luciferin
NTO: non‐transcriptional oscillator
PER: period
TBB: tetrabromo‐2‐azabenzimidazole
TFA: trifluoroacetic acid
THP: tris (hydroxypropyl) phosphine
TOC1: timing of Cab2 expression 1
TOST: two one‐sided test
TTFL: transcriptional/translational feedback loop
WCC: white collar complex
ZT: Zeitgeber time

## 1 Introduction

Throughout evolution, life on our rotating planet has been exposed to the sun in a predictably rhythmic fashion, and most of current life found on earth exhibits a potential for 24‐h timekeeping by an endogenous, circadian clock. Some outputs of these clocks can be observed with the naked eye, such as diurnal rhythms in animal behaviour or the rhythmic orientation of plant leaves to the sun. These rhythms result from cellular circadian clocks that drive a cycle of transcriptional reprogramming every day. Rhythmic transcription and translation of clock genes in turn generates the oscillation directly or indirectly via a network of feedback loops, and oscillations of these clock transcription factors initiate rhythms in a large number of clock‐controlled genes 1. The role of post‐translational control of these clock proteins is crucial to tune the genetic networks into a ∼24 h rhythm, nudging the focus of research in chronobiology away from genetics and into biochemistry 2, 3.

**Significance of the study**

Rotation of planet Earth generates a diurnal cycle of day and night. Nearly all eukaryotic taxa have evolved intricate cell‐autonomous clockworks to anticipate this daily rhythm. Although the components of transcriptional clock networks are different across higher taxa, the regulatory action of post‐translational regulator proteins such as casein kinase 2 (CK2) is functionally conserved in all eukaryotic kingdoms. An extensive list of potential CK2 target sites in the minimal model eukaryote *Ostreococcus tauri* (*O. tauri*) is reported here, and because many of the corresponding mother proteins are highly conserved this list might harbour important answers to how CK2 can be involved in timekeeping across such diverse organisms with such diverse clock networks. Results from this fundamental research are therefore directly relevant to applied research, including crop and medical sciences.

CK2 is a holoenzyme, consisting of a tetramer of two regulatory beta subunits and two catalytic alpha subunits, which regulates a variety of cellular processes 4 including the rhythmic processes of the circadian clock and the cell cycle 5; it is therefore a key target in anti‐cancer pharmacology 6. CK2 homologs are present in all eukaryotic life and so far, CK2 has been shown to affect circadian clock period length in mammals 5, flies 7, 8, fungi 9, 10, and plants 11, 12, 13, 14. If the role of CK2 in the circadian clock is conserved across these species, a plausible explanation would be that unidentified, conserved targets exist. To efficiently study the role of CK2 in timekeeping, it would be beneficial to study prototypical eukaryotes. The *O. tauri* cell is the smallest free‐living eukaryote 15, containing only one mitochondrion, Golgi stack, and chloroplast. All the information to operate these cells is encoded by only ∼8000 genes 16. 133 protein kinase genes found in *O. tauri* represent all major clades except the receptor‐like kinases and genuine tyrosine kinases 17. Both CK2 subunits are encoded by a single gene, which is very strongly related to the human and other animal homologs. The *O. tauri* circadian clock has been studied in some detail both in vivo 18, 19, 20 and in silico 21, 22. Whilst the simplicity of these cells is key to the efficient study of eukaryotic signalling, it does provide a challenge to obtain sufficient material for quantitative proteomics; cells divide a maximum of once a day, are tiny, and do not grow to high densities. In spite of this challenge, workable quantitative phosphoproteomic methods have previously been established to study the roles of another conserved protein kinase, casein kinase 1 (CK1) 23, 24, which is structurally unrelated to CK2. Therefore, *O. tauri* offers the ideal background to efficiently study canonical CK2 signalling in the circadian system and other conserved processes.

Overexpression of CK2 did not lead to major changes to the overall proteome, but clearly revealed functional conservation in the timekeeping mechanism in this organism. Subsequent analyses of the phosphoproteome in wild‐type cells, CK2‐overexpressing cells, and CK2‐inhibited cells then revealed a total of 3058 phosphopetides, corresponding to 1520 unique phosphopeptide motifs. Comparisons between treatments resulted in a list of 447 unique CK2‐responsive phosphopeptide motifs in this minimal eukaryotic background. This catalogue of CK2 targets is a rich resource to inform studies in more complex organisms, and reveal additional roles of CK2 in human health and other economically relevant areas.

## 2 Materials and methods

### 2.1 Culturing and luminescent imaging

All chemicals and reagents were ordered from Sigma‐Aldrich (UK), unless otherwise stated. Cells were cultured as described in 19, 25. Luminescent CCA1‐LUC cells are published elsewhere 18. For imaging experiments, cells were transferred to white microplates (Lumistar, Greiner BioOne, UK) one week before the start of imaging, and luciferin (LUC) was added to 2.5 mM in a medium change one day before the start of imaging on a TopCount plate reader, as described in 20. Luminescent traces were imported in BRASS v3.0 26 software, and all period lengths were analysed by the mfourfit algorithm. For washout experiments, 5,6,7‐tetrabromo‐2‐azabenzimidazole (TBB) was added (5 μM) to eight replicate wells at the given ZT, and washout was achieved by removing all media from the naturally formed cell aggregates in the bottom of each well using a multichannel pipette.

### 2.2 Overexpression of CK2

A Gateway‐compatible version of the overexpression vector POTOX 18 was previously reported 23. CK2 alpha was amplified from *O. tauri* genomic DNA (aaaaagcaggctacATGTCCACGCGCATCGGTAAGG and agaaagctgggtaGAACCAAGACGTTTCGAGTGTTAG) and cloned into pDONR207 using Gateway technology (Invitrogen). Vectors were sequenced and transformed into the CCA1‐LUC background by methods published previously 18, 25. Successful transformation was tested by two rounds of selection on ClonNat (Werner Biochemicals, Germany) and PCR amplification of the selectable marker from DNA isolated from transformed lines. CK2 levels upon overexpression were tested by qRT‐PCR using CK2 oligo's GGCGATTGATTTCCTCGATA and AAAAGAAGCGAGGAGGGAAG compared to elongation factor 1a as a standard (CCAGGCGGACGCCGGAATTT and TCGACGATGTGTTGAAAACG). qPCR results were verified with label‐free quantitative mass spectrometry as described below.

### 2.3 Protein extraction and digestion

*O. tauri* cells were lysed and prepared as described previously 23. For CK2 inhibition, a concentration of 1 μM TBB was chosen since a maximum circadian period increase was observed at this concentration 20. Drugs were added 24 h before harvest. *O. tauri* cell pellets were resuspended in 8 M urea and disrupted by sonication. 300 μg of protein extract was reduced in 5 mM tris(hydroxypropyl)phosphine (THP) for 30 min, and cysteines were alkylated in 10 mM iodoacetamide. Samples were diluted to 2 M urea in ammonium bicarbonate buffer (25 mM). Trypsin (modified, sequencing grade, Roche, UK) was added (1:50) for overnight digestion. Reactions were stopped with 2% formic acid. Peptide digests were cleaned up using a SupelCleanC18 cartridge and dried under low pressure. A fraction (10 μg) was kept for direct LC‐MS analysis, and samples were stored at –20°C.

### 2.4 Phosphopeptide enrichment

Peptides were dissolved in 2.5% acetonitrile (ACN) and 0.5% trifluoroacetic acid (TFA), and mixed with an equal volume of 80% ACN, 200 mg/ml 2,5‐dihydroxybenzoic acid (DHB) and 0.5% TFA. Phosphopeptides were enriched from 300 μg total peptide extract using in‐house built titanium dioxide microcolumns (4 × 0.4 cm, 10 μm in diameter; Titansphere, GL Sciences, Japan), manually operated using a pressure vessel. Columns were washed with (80% ACN, 200 mg/ml DHB, 0.1% TFA), followed by (80% ACN, 0.5% TFA), and (80% ACN, 0.1% TFA). Phosphopeptides were eluted using 400 mM ammonium, then 5% (v/v) ammonium, then 5% (v/v) ammonium in ACN. Pooled elutions were dried under low pressure and stored at –20°C.

### 2.5 Nano‐LC‐MS/MS

Nano‐liquid chromatography tandem Mass Spectrometry analyses were performed using a micro pump (Agilent 1200 binary HPLC system, Agilent Technologies, UK) coupled to an LTQ‐Orbitrap XL hybrid mass spectrometer (ThermoFisher, UK) as described previously 27. MSMS data were searched using MASCOT Version 2.4 (Matrix Science Ltd, UK) against the *O. tauri* subset of the NCBI protein database (12/01/2011; including common contaminants) using a maximum missed‐cut value of 2, variable oxidation (M), N‐terminal protein acetylation, phosphorylation (STY) and fixed carbamidomethylation (C); precursor mass tolerance was 7 ppm and MSMS tolerance 0.4 amu. The significance threshold (*p*) was set below 0.05 (MudPIT scoring). Global FDR was evaluated using decoy database search and removal of peptides ranked higher than 1 for a mascot score above 20 (∼1% global FDR). The mass spectrometry proteomics data have been deposited to the ProteomeXchange Consortium 28 via the PRIDE partner repository with the dataset identifier PXD000975 and DOI 10.6019/PXD000975. Data were converted into PRIDE‐XML using Pride converter 2.0.20 29 and submitted using proteome exchange tool px‐submission‐tool 2.0.1 30.

### 2.6 Data analysis

Label free quantitation was performed using Progenesis 4.0 (Nonlinear Dynamics, UK) as described previously 27. All multicharged ions (2+, 3+, 4+) were extracted and ion intensities summed for normalization. Peptide abundances were mean‐normalised and arcsinh transformed to generate normal datasets for pairwise *t*‐test calculations. Within‐group means were calculated to determine fold changes. For over‐ and under‐representation of peptide–motifs we required a two‐tailed *t*‐test *p*‐value of < 0.05 and an absolute fold change ratio of > 1.5. For unchanging motifs we required a two one‐sided test (TOST) with a *p*‐value of < 0.05 and an absolute fold change ratio of < 1.5. Global *p*‐value evaluation was performed using Progenesis, and pair‐wise *t*‐tests were performed in R (3.1.0). GO term enrichment was evaluated using STRING (http://string‐db.org/). Phosphopeptide motif assignment was confirmed using Maxquant 31. Peptide intensities were merged in a motif‐like manner; multiple intensity values for the same phosphorylated peptide motif with different charges, incomplete protease digestions, and oxidised variants were summed to generate a single abundance value. pLogo version 1.2.0 32 was used to detect motif patterns that were significantly overrepresented (*p* < 0.05) within each significance group compared to the whole proteome background. We extended each peptide motif detected to a 15 amino acid window. To include all detected peptide motifs, we used X to represent overlap (background terminal peptide sequences were extended with a seven amino acid X region to remove statistical significance of X from the motif logo). Overlap between significance sets in the CK2 overexpression and inhibition datasets were visualised using the chord visualization diagram 33 from the D3 library (http://d3js.org).

## 3 Results

### 3.1 Overexpression of CK2 leads to long period of the circadian rhythms

**Figure 1:**
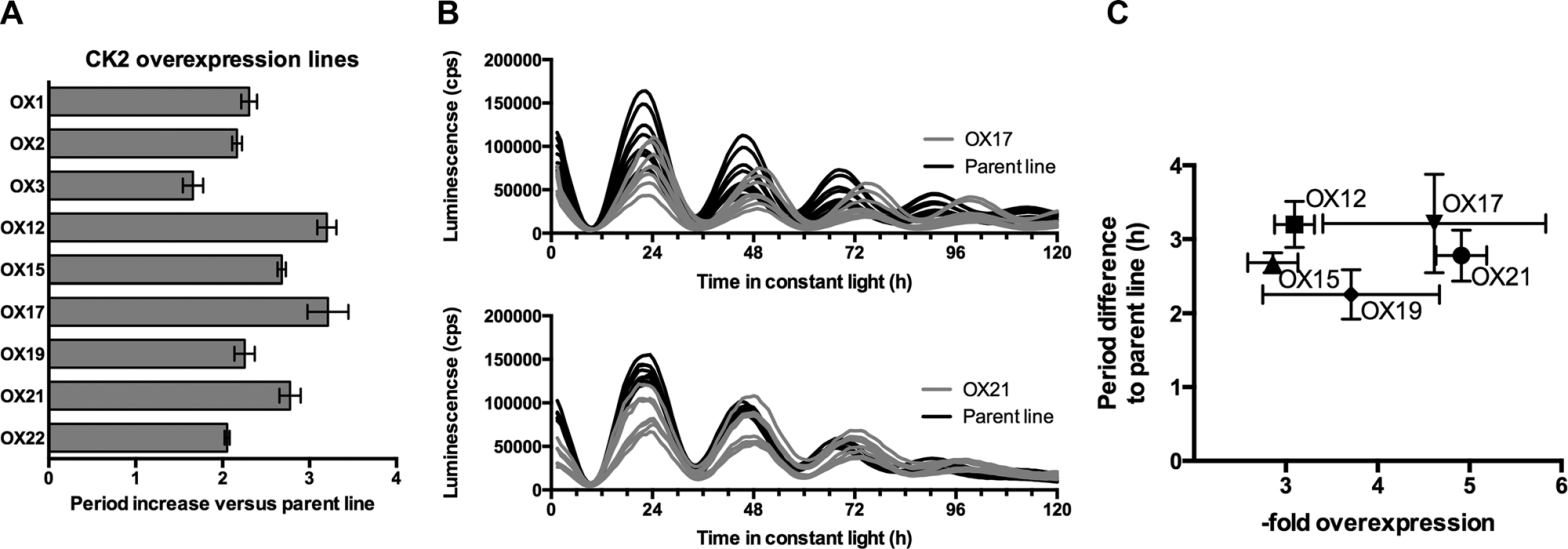
CK2 overexpression leads to an increase in circadian period length. **(**A) A CK2 overexpression construct was transformed into the rhythmically luminescent parent line CCA1‐LUC. Free‐running period was analysed by luminescent imaging in all nine positively transgenic CK2‐OX lines. Period length relative to the parent line is indicated. In all cases, a significantly (*p* < 0.001, *n* = 8) long circadian period was observed. (B) Two examples showing the eight individual replicate luminescent traces of overexpression lines CK2‐OX17 (top panel) and CK2‐OX21 (bottom panel) in grey, compared to the parent line CCA1‐LUC in black. (C) Period increase of the overexpression lines plotted against overexpression levels as analysed by qRT‐PCR of the transgene. Error bars reflect standard deviation based on three (*x*‐axis) or eight replicates (*y*‐axis).

The transcriptional clock in *O. tauri* contains a central loop between the clock components timing Of Cab2 expression 1 (TOC1) and circadian clock associated 1 (CCA1). Luminescent marker lines were reported previously where the rhythmically regulated CCA1 promoter drives the expression of CCA1 protein fused to firefly luciferase (CCA1–LUC) 18. The CCA1–LUC line was transformed with an overexpression construct of the CK2 catalytic subunit alpha to allow investigation of circadian changes in the transcriptional clock network reported by the CCA1 promoter. The circadian free‐running period of nine independent overexpression lines (CK2‐OX) was analysed in constant light (LL). A slow circadian clock was observed, up to a period increase of ∼3 h (Fig. 1A, B) compared to the parent line. In all lines, a significant overexpression of the CK2 mRNA was observed by qRT‐PCR (Fig. 1C). This result indicates that the role of CK2 in circadian timekeeping is functionally conserved from animals and fungi to the unicellular alga *O. tauri*.

### 3.2 The effect of CK2 overexpression on the global proteome

To detect the effect of CK2 overexpression on the global proteome, parent line CCA1–LUC was compared to the CK2‐OX21 line (*n* = 5) using nano‐LC‐MS/MS at two phases of the day. Time‐of‐day is indicated in Zeitgeber time (ZT) where dawn is defined as time 0 (ZT0) and, in a 12:12 light/dark cycle, dusk as ZT12. In the combined dataset of the resulting 20 nano‐LC‐MS/MS runs, a total of 1292 proteins were identified (Supplemental Table 1).

**Figure 2:**
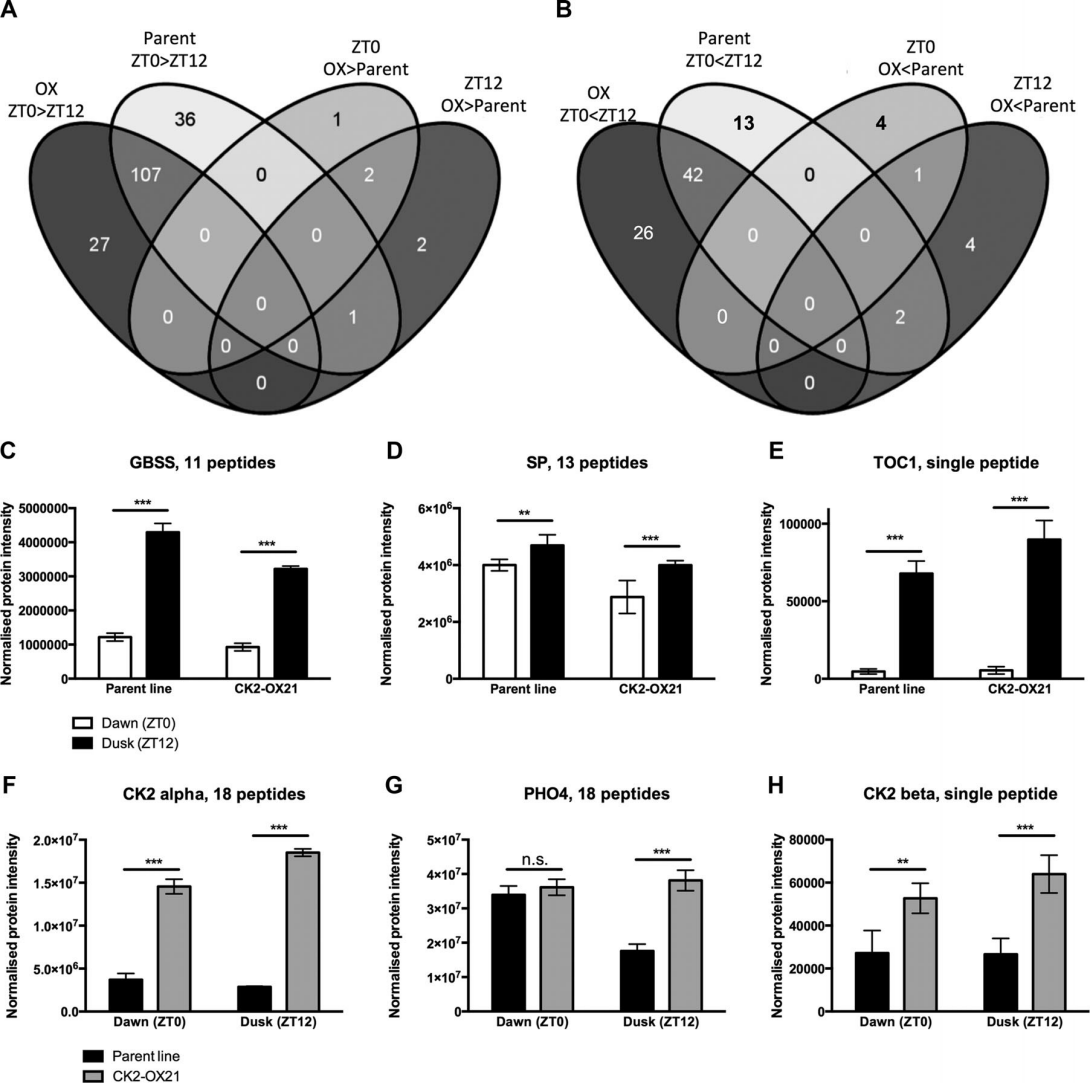
Effects of CK2 overexpression on the global proteome at dusk and dawn. The global proteome at dawn (ZT0) was compared to that at dusk (ZT12) in the CK2‐OX21 and parent lines using nano‐LC‐MS/MS. The abundance of proteins represented by at least two peptides were compared between treatments and a venn diagram was generated from those sites upregulated (A) or downregulated (B) in one treatments versus the other. Examples of diurnally regulated proteins are provided in panels C‐E, and proteins of differential abundance in the overexpression line in panels F‐H. (C) Granule‐bound starch synthase 1 (GBSS, represented by eleven quantified peptides). (D) Starch phosphorylase (SP, 13 peptides). (E) The TOC1 protein (APRR‐like), represented by a single peptide for which the MS/MS spectra are provided in the supplemental data. (F) The overexpressed CK2 alpha subunit, represented by 18 quantified peptides. G) High‐affinity phosphate transporter (PHO4, 18 quantified peptides). C) The CK2 beta subunit, only represented by a single peptide, for which the MS/MS spectra are provided in the supplemental data. Error bars represent standard deviation of five replicates. *** indicates a *p*‐value of < 0.005 (pairwise *t* tests on arcsinh transformed data). **: *p*‐value < 0.05. The total dataset of all quantified proteins can be found in Supplemental Table 1.

Differences in protein quantities between the two lines and/or the two time points were analysed by pair‐wise *t*‐test (Supplemental Table 1). A summary of all proteins upregulated (Fig. 2A) or downregulated (Fig. 2B) compared between timepoints or cell line reveals that the global proteomic changes between between timepoints is far larger than between the parent and CK2‐OX line (*p* < 0.05, fold change > 1.5). In photosynthetic organisms, the difference between night and day constitutes a major change to cellular metabolism. Previously observed circadian oscillations in cellular redox metabolism 20 are reflected by a GO terms enrichment (*p* = 1.44e‐3) of 18 proteins involved in oxidation/reduction from the pool of proteins (107 in total) that are significantly more abundant at dawn than at dusk in both lines. Among the 36 proteins whose upregulation in the parent line is lost in CK2‐OX21, hexose metabolism is overrepresented (three proteins, *p* = 3.60e‐2). In the set of 27 proteins uniquely upregulated at ZT0 in the overexpression line, an enrichment (seven proteins, *p* = 5.52e‐3) of proteins involved in translation is observed. Some specific examples of differential protein levels are provided in Fig. 2.

Differential regulation of starch metabolism is of particular importance to anticipate the daily light/dark cycle; during the photosynthetic phase starch is produced in plants, whilst starch degradation provides the sugar supply for metabolism during the night 34. A diurnal output rhythm in starch metabolism is indeed observed here in *O. tauri*; a granule‐bound starch synthase protein (GBSS, Fig. 2C) is significantly more abundant at the end of the day (ZT12), compared to the end of the night (ZT0). A starch phosphorylase (SP, Fig. 2D), important to break down starch during the night, is upregulated at dusk in anticipation of the night ahead. Variations between the dusk and dawn time points are in agreement with a previous study in cases where a protein was quantified in both, for example the peak at ZT12 in GBSS (Fig. 2D and 27).

Clock components themselves are differentially abundant through a day, but are notoriously lowly abundant. The absolute number of molecules of TOC1 per cell was previously estimated to oscillate between peak and trough levels of 150 and 10 proteins per cell 19. Given this low abundance, it is noteworthy that a single peptide (MS/MS spectra are provided in Supplemental Fig. 1B) derived from the TOC1 protein was detected (Fig. 2E). In line with the evening phase of TOC1 expression, this peptide was upregulated at dusk compared to dawn both in wild‐type and CK2‐OX21. The observed fold change of > 19 is remarkably similar to the previously reported 15‐fold change obtained using totally independent methodology 19.

In addition to differential levels of protein abundance between timepoints, a few significant changes are associated with CK2 overexpression. Firstly, CK2 itself is an abundant protein that was identified with at least 18 peptides. Consistent with the overexpression observed on the RNA level (Fig. 1C), the level of CK2 protein in CK2–OX21 was strongly increased compared to the parent line CCA1–LUC (maximum 6.5 fold change, Fig. 2F), verifying successful overexpression. Additional significant changes in protein abundance result from CK2 overexpression, and an interesting example is the high‐affinity phosphate transporter, PHO4. In the parent line, a diurnal protein expression level is evident, whereas in the CK2–OX21 line, PHO4 expression is at high levels regardless the diurnal phase (Fig. 2G). Phosphate transporter expression in *O. tauri* is regulated by cellular demand for phosphate 35, implying an increased demand for phosphate at that phase upon CK2 overexpression, compared to a lower demand in wild‐type cells. Thirdly, based on a single peptide (MS/MS spectra in Supplemental Fig. 1B) an approximate two‐fold upregulation of the regulatory subunit CK2 beta is evident at both timepoints (Fig. 2H) in CK2–OX21, though only the catalytic kinase subunit of the holoenzyme is overexpressed here. This result indicates that cells have adjusted to the elevated levels of CK2 alpha subunits by upregulation of the beta subunit.

### 3.3 Phase‐dependent role in timekeeping

**Figure 3:**
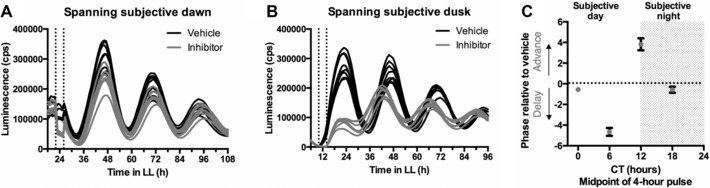
CK2 contributes to timekeeping phase‐dependently. The effects of pulsed treatments with CK2 inhibitor TBB was analysed on the luminescent reporter line CK2‐LUC. Cells were treated to 4‐h pulses of inhibition in the middle of subjective day (from 4–8 h into LL), around subjective dusk (10–14 h), in the middle of subjective night (16–20 h) and around subjective dawn (22–26 h). Examples of traces after washout of the drugs are provided for treatments spanning subjective dawn (A) and dusk (B). The phase of the first peak of the resulting circadian rhythms was analysed for all treatments and plotted on a phase‐response curve (C).

Contributions of CK2 activity on wild‐type timekeeping could be phase‐dependent if phosphorylation of clock‐relevant target proteins is clock‐gated. To test this hypothesis, the effect of 4‐h pulsed inhibition of CK2 using the pharmacological agent TBB 20, 36 was compared across different times of the day (Fig. 3). Cells were transferred to constant light (LL) to assess circadian‐over diurnally‐regulated effects. ‘Dawn’ and ‘dusk’ are therefore subjective in this experiment, as actual light/dark transitions do not exist. Treatments spanned either subjective dusk or dawn, or the middle of the subjective day or night. The effect of the treatments was monitored by luminescent imaging following washout of the drugs (*n* = 8). Clear circadian rhythmicity is resumed after the inhibition pulse, verifying efficient washout of the inhibitor. A pulse during subjective dawn did not alter the phase of subsequent rhythms (Fig. 3A), but a large phase advance of the resulting rhythm is observed after inhibition spanning subjective dusk (Fig. 3B). Phase changes induces by all four treatments are plotted on a circadian phase‐response curve (Fig. 3C). These results suggest a larger contribution of CK2 activity on cellular timekeeping around dusk than around dawn.

### 3.4 Phosphoproteome analyses

In addition to the global proteomics (Fig. 2), extensive phosphoproteomic analyses were performed at ZT12, to maximise the potential identification of clock‐relevant CK2 target proteins based on the pharmacologically identified contribution of CK2 to timekeeping around dusk. Protein extractions harvested at this phase were trypsinated, enriched for phosphopeptides, and analysed by mass spectrometry. We compared the parent line phosphoproteome to that of the CK2 overexpression line CK2–OX21, and to that of cells treated with CK2 inhibitor TBB (*n* = 5 for all). Throughout these experiments, 3058 phosphopeptides were observed (Supplemental Table 2). These phosphopeptides included different variants of the same phosphopeptide motif due to charge variants, incomplete trypsin digestion, and oxidation variants. Therefore, multiple intensity values of the same phosphorylated peptide motif were merged into one single abundance value per unique phosphopeptide motif. This analysis revealed that the 3058 observed peptides corresponded to 1520 unique phosphopeptide motifs (Supplemental Table 2 provides lists of peptides pre‐ and post‐merging). Upregulation of phosphopeptide motifs cannot be caused by higher expression in the CK2 overexpression, as no major proteomic changes are observed (Fig. 2A, B) in the overexpression line compared to the parent line (only 14 proteins with differential abundance are observed). For example, a phosphopeptide motif on the putative ATPase Ot01g03680 was strongly upregulated upon CK2 expression (Supplemental Table 2), whereas the actual protein level quantified over eleven unique peptides does not vary significantly (Supplemental Table 1). To identify the consequences of modified CK2 activity, we compared the quantitative phosphoproteomic profiles of treated versus control cells.

### 3.5 Phosphoproteomics I: effects of CK2 overexpression

**Figure 4:**
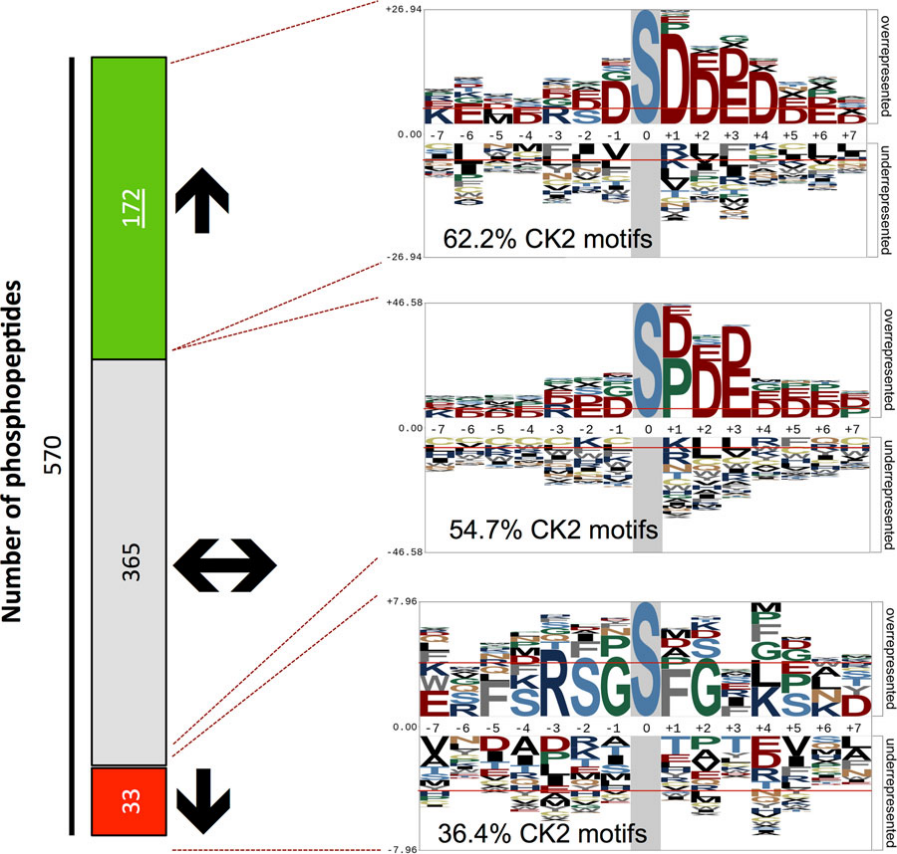
Distribution of potential CK2 sites among significantly quantified phosphopetides. Comparison of significant pLogo motifs identified in the indicated groups of phosphopeptides upon CK2 overexpression compared to the parent line (log‐odds of the binomial probability on *y*‐axes). Green: significantly upregulated peptides (é). Grey: peptides with significantly equal mean. Red: significantly downregulated peptides (ê). Percentages of sites that fit predicted CK2 motifs are given for each pLogo group. Combined CK2 motifs are: S/T‐D/E, S/T‐X‐D/E, S/T‐X‐D/E‐D/E, S/T‐D/E‐X/D/E, S/T‐X‐X‐D/E, AND S/T‐D/E‐D/E‐D/E, whereby X denotes any amino acid.

The large number of phosphopeptide motifs differentially abundant in the CK2–OX line compared to the parent line are visualised on a volcano plot (Supplemental Fig. 2A). In line with the overexpression of a prolific kinase such as CK2, a clear general trend can be observed towards upregulation of phosphopeptide motifs over downregulation. Comparing the overexpression line to the parent line generates three classes of significantly quantified phosphopeptide motifs; those that are significantly upregulated (total 172, Fig. 4) or downregulated (total 33) (*p* < 0.05, fold change > 1.5, two‐tailed *t*‐test), and those that are statistically unchanged in mean abundance (total 365, *p* < 0.05, fold change < 1.5, two one‐sided test) between the overexpression and parent line. Those motifs that were not confidently within one of these groups were omitted from the analyses as described in the methods section. pLogo enrichment of the amino acid sequences around the phosphorylated sites was analysed for the three significant groups separately (Fig. 4). Direct targets of CK2 would be expected in the upregulated set of motifs, and indeed 62.2% of upregulated motifs conformed to the defined CK2 target site defined. In the statistically unchanged phosphopeptide motifs, potential CK2 target sites are also highly abundant (Fig. 4 middle panel), possibly reflecting sites that are already highly phosphorylated in wild‐type cells given the prolific activity of CK2 4. An interesting difference between the non‐differential group and the upregulated group is the significant enrichment of a proline in position + 1 in the former. This result suggests that other abundant kinase activities (i.e. proline‐directed kinases like CK1 and GSK3) are not differentially affected by CK2 overexpression. In the down‐regulated set of phosphopeptide motifs, CK2 sites should be depleted and secondary phosphorylation events associated with knock‐on effects of CK2 overexpression would be expected. Indeed, a reduced number (36.4%) of CK2 sites are identified in the downregulated set compared to the non‐differential set (Fig. 4, lower panel). Technically, this result shows that the enrichment of acidic residues at +/– 7 position in the other two groups did not result from a bias associated with our TiO_2_ enrichment methods. Biologically, this result could indicate that down‐regulated sites derive from proteins potentially involved in crosstalk or feedback within the kinase network, as they reveal CK2‐mediated changes in other kinase activities.

### 3.6 Phosphoproteomics II: effects of the CK2 inhibitor TBB

Pharmacological inhibition of CK2 was previously shown to increase circadian period length 20. To further characterise the effects of CK2 on the total phosphoproteome and to identify additional potentially clock‐relevant target sites, pharmacologically inhibited cells were compared to control cells, harvested at ZT12. Similar to when comparing the overexpression line to the parent line (Fig. 4), comparing inhibitor‐treated cells to untreated cells generates three classes of significantly quantified phosphopeptide motifs; significantly upregulated motifs, significantly downregulated motifs, and motifs with statistically significantly unchanged mean abundance between treated and non‐treated cells. Motifs that were not quantified with statistical significance were omitted from the analysis. Fold‐changes and *p*‐values of all unique phosphopeptide motifs are provided in Supplemental Fig. 2B and Supplemental Table 2.

**Figure 5:**
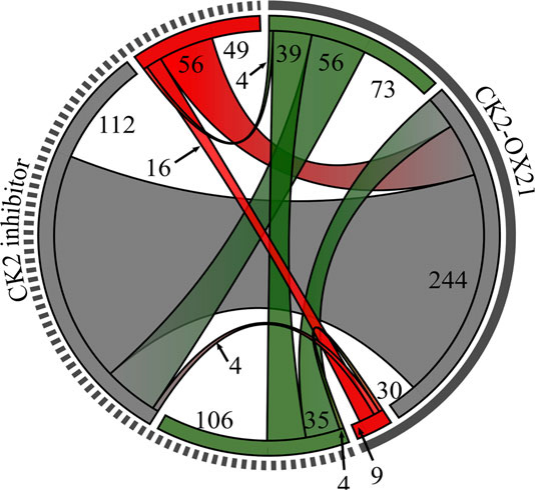
Overlap between significant groups of phosphopeptide motifs upon CK2 overexpression or inhibition relative to controls. Significantly quantified groups of phosphopeptide motifs that were upregulated (green), downregulated (red), or non‐differential (grey) compared to the control cells upon CK2 overexpression or inhibition are compared side by side (labelled on the circumference). Shared chords represent overlap between the six different groups, and numbers of phosphopeptide motifs in each unique group or shared chords are provided.

Comparison of unique phosphopeptide motifs in the three significantly quantified groups following overexpression or inhibition allows visualisation of potential overlap between any of the six groups (Fig. 5). Numbers of significantly quantified but non‐differential sites (grey), upregulated sites (green), and downregulated sites (red) upon either overexpression or inhibition of CK2 are plotted opposite each other, with chords indicating overlap between each significant group. The majority of the phosphopeptide motifs would be expected to be unaffected by changes to cellular levels of CK2 activity. Indeed, many phosphopeptide motifs (244) that were significantly unchanged in response to either treatment were detected in both experiments, meaning that base levels of phosphorylation on these sites are observed at this phase. A large proportion of sites upregulated upon overexpression are non‐differential upon TBB treatment. These 56 sites could represent CK2 activity that is not normally present at the dusk phase in wild‐type cells, and would thus be equally low in control versus inhibited cells. An identical overlap of 56 sites is observed between sites that are downregulated upon inhibition but not responsive to overexpression. No significant GO terms enrichments are identified in individual chords, and these sites could either represent secondary or off‐target effects of the drug, or sites that are normally already highly phosphorylated at the dusk phase, and thus not responsive to further increases in CK2 levels in the overexpression line.

## 4 Discussion

Whereas the transcriptional loops behind oscillating transcripts and their products are reasonably well understood as a result of decades of good work by the international community, the post‐translational regulation of these clock proteins is not well mapped. The observation that CK2 affects the period length and clock function in an array of organisms with a distinctly separate group of TTFL components 5, 7, 10, 36, implies that additional CK2 targets or functions might exist that explain the striking functional conservation of CK2 activity in cellular timekeeping. The significant changes to the global and phosphoproteome upon modulation of CK2 activity reported here, are therefore likely to contain conserved CK2 targets with relevance to timekeeping or CK2 networks in higher taxa.

A priori, a large overlap would be expected between those sites upregulated by CK2 overexpression and those downregulated by CK2 inhibition. However, the anticipated overlap between sites upregulated following overexpression and downregulated by inhibition is remarkably small (four sites). Instead, results indicate that each treatment alters the abundance of a generally different set of phosphopeptide motifs, indicates that there is mileage in combining the two strategies to identify a maximum number of potential CK2 target proteins.

Explanations to why both treatments affect different sets of peptides are (i) substrate specificity effects resulting from the overexpression of a catalytic kinase without its regulatory subunit, and (ii) complex network interactions within the kinase network. The former is unlikely to fully explain our results, as the observed overexpression of the catalytic subunit (3‐ to 5‐fold) is partly matched by upregulation of the regulatory subunit (Fig. 2H). The latter is a more likely source of complexity, as a number of kinase proteins are differentially phosphorylated in the overexpression line as well as in the CK2‐inhibited cells (Supplemental Table 2). These modifications could impact on the activity or selectivity of these kinases, complicating the separation of direct from indirect effects on the phosphoproteome. Interestingly, a phosphopeptide motif on a calcium‐dependent protein kinase (Ot01g01760) is one of only three phosphopeptide motifs among those upregulated by CK2 overexpression that was also upregulated upon overexpression of the unrelated but equally conserved clock kinase CK1 23. Given that the kinase network is an intertwined complex network 37, the knock‐on effects of perturbation are challenging to predict (or indeed explain). Importantly, it was previously reported that inactivation of most kinases or phosphatases did not only affect the direct targets of those proteins, but in fact large portions of the phosphoproteome 38.

Oscillatory systems, like the cell cycle or the circadian clock, create another layer of complexity as the kinase, the target, and probably also the phosphatase, are differentially abundant through a circadian day. A number of complex behaviours of kinase networks in these oscillatory cellular systems have been described, like hysteresis or multispecificity (cf. 39, 40, 41, 42, 43, 44. Taken together, complex behaviour of the kinase network should have been the null hypothesis all along, as our results once again confirm the need for applying mathematical power to help the human brain making sense of complex systems biology questions.

CK2 and other post‐translational regulator proteins affect the circadian clock in a diverse set of organisms, which is surprising because the classical clock components in these species are structurally unrelated. Our current understanding of cellular CK2 activity in the clock generally derives from complex multicellular organisms or tissues. To efficiently study basic timekeeping, it is beneficial to study prototypical eukaryotes that generate robust and complete circadian rhythms from minimal genetic and cellular complexity. Even in the reduced model organism *O. tauri*, we now report unanticipated complex behaviour of the kinase network in response to modified CK2 activity. The results reported here will therefore act as a starting point to unravel conserved roles in timekeeping for CK2 targets across eukaryotes.

## Supporting information

Figure S1. MS/MS spectra for those proteins in Figure 2 quantified based on a single peptide.

Figure S2. Volcano plots of mean phosphopeptide quantifications upon CK2 overexpressions (A) or inhibition (B) compared to control cells.

Supplementary Table 1. listing identified peptides with identification and quantification details

Supplementary Table 2. listing identified peptide, phosphopeptides and phospho‐motifs with identification and quantification details
