# Supplementary figures and images for: Label‐free quantitative analysis of the casein kinase 2‐responsive phosphoproteome of the marine minimal model species Ostreococcus tauri
Source: Proteomics. 2015 Jun 9;15(23-24):4135–44. doi: 10.1002/pmic.201500086 (PMC4716292; doi:10.1002/pmic.201500086)

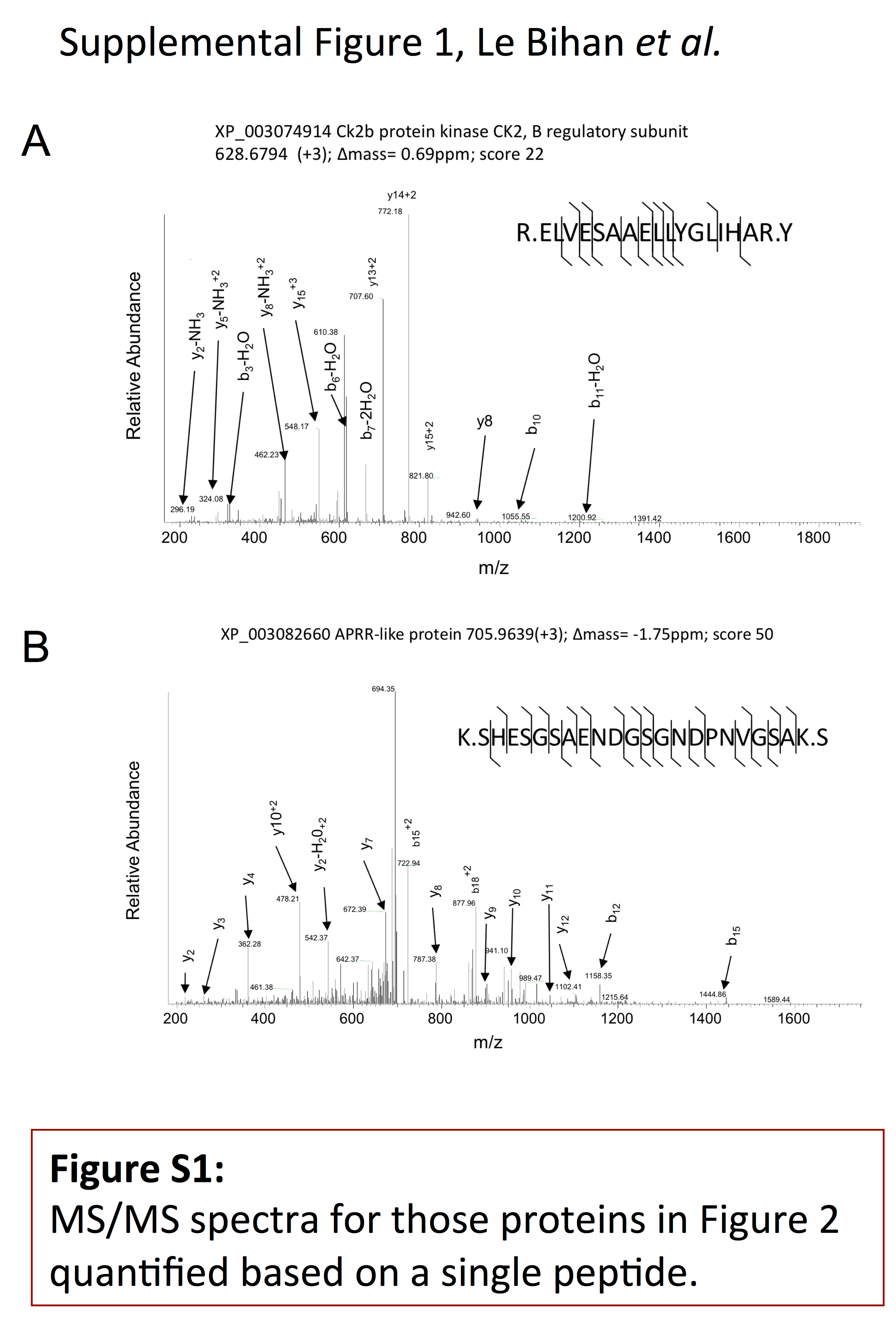

Supplement: Supplementary file 1 — Figure S1. MS/MS spectra for those proteins in Figure 2 quantified based on a single peptide. [file PMIC-15-4135-s001.tiff]

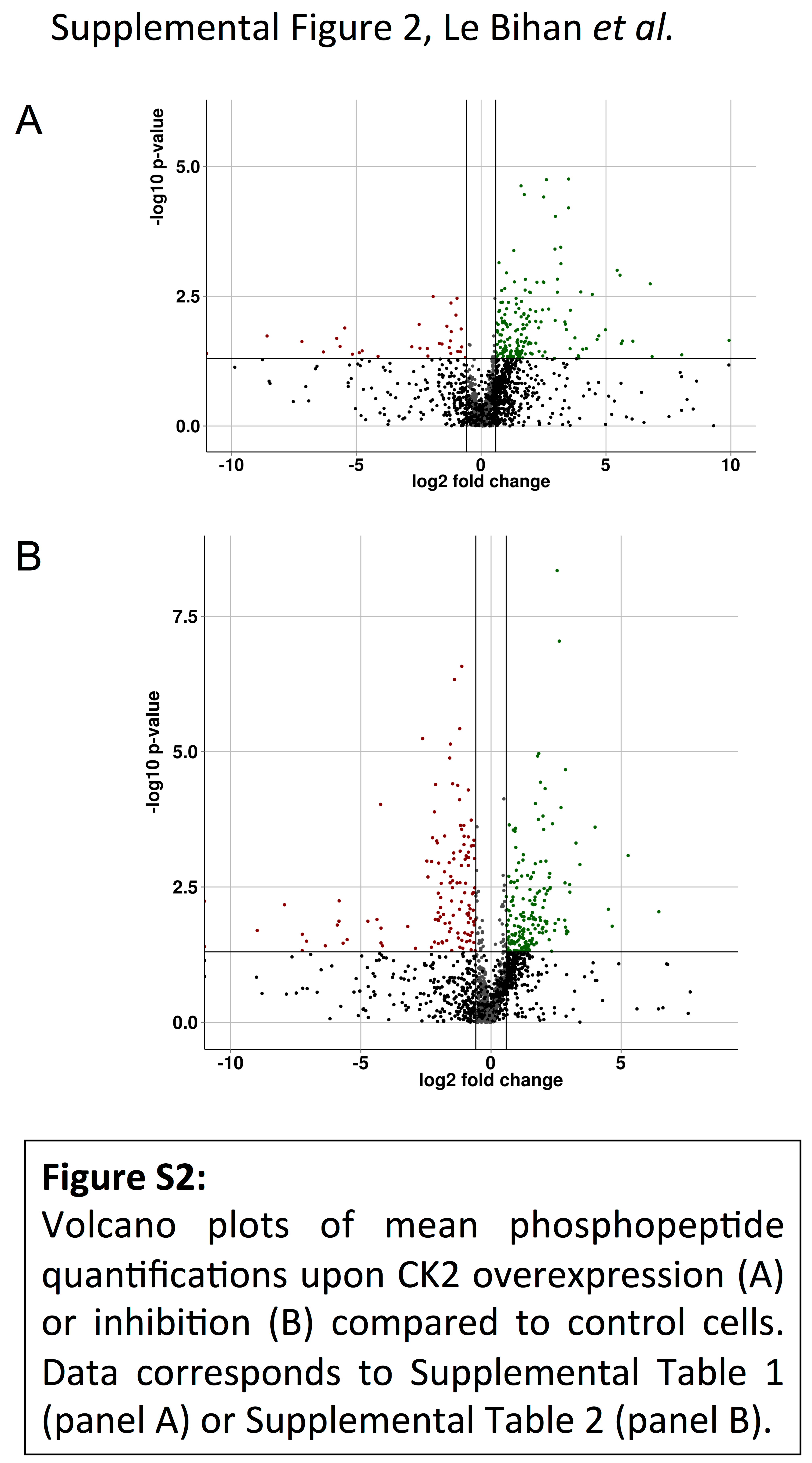

Supplement: Supplementary file 2 — Figure S2. Volcano plots of mean phosphopeptide quantifications upon CK2 overexpressions (A) or inhibition (B) compared to control cells. [file PMIC-15-4135-s002.tiff]
